# Cystic Fibrosis Sputum Impairs the Ability of Neutrophils to Kill *Staphylococcus aureus*

**DOI:** 10.3390/pathogens10060703

**Published:** 2021-06-04

**Authors:** Kayla Fantone, Samantha L. Tucker, Arthur Miller, Ruchi Yadav, Eryn E. Bernardy, Rachel Fricker, Arlene A. Stecenko, Joanna B. Goldberg, Balázs Rada

**Affiliations:** 1 Department of Infectious Diseases, College of Veterinary Medicine, The University of Georgia, Athens, GA 30602, USA; kayla.fantone25@uga.edu (K.F.); sltucker@uga.edu (S.L.T.); arthur.miller@uga.edu (A.M.); ryadav@uga.edu (R.Y.); rachel.fricker25@uga.edu (R.F.); 2 Division of Pulmonology, Allergy/Immunology, Cystic Fibrosis and Sleep, Department of Pediatrics, Emory University School of Medicine, Atlanta, GA 30322, USA; ebernardy@elon.edu (E.E.B.); astecen@emory.edu (A.A.S.); joanna.goldberg@emory.edu (J.B.G.)

**Keywords:** cystic fibrosis, PMN, *Staphylococcus aureus*, killing, sputum, respiratory burst, neutrophil extracellular traps

## Abstract

Cystic fibrosis (CF) airway disease is characterized by chronic microbial infections and infiltration of inflammatory polymorphonuclear (PMN) granulocytes. *Staphylococcus aureus (S. aureus)* is a major lung pathogen in CF that persists despite the presence of PMNs and has been associated with CF lung function decline. While PMNs represent the main mechanism of the immune system to kill *S. aureus*, it remains largely unknown why PMNs fail to eliminate *S. aureus* in CF. The goal of this study was to observe how the CF airway environment affects *S. aureus* killing by PMNs. PMNs were isolated from the blood of healthy volunteers and CF patients. Clinical isolates of *S. aureus* were obtained from the airways of CF patients. The results show that PMNs from healthy volunteers were able to kill all CF isolates and laboratory strains of *S. aureus* tested in vitro. The extent of killing varied among strains. When PMNs were pretreated with supernatants of CF sputum, *S. aureus* killing was significantly inhibited suggesting that the CF airway environment compromises PMN antibacterial functions. CF blood PMNs were capable of killing *S. aureus*. Although bacterial killing was inhibited with CF sputum, PMN binding and phagocytosis of *S. aureus* was not diminished. The *S. aureus*-induced respiratory burst and neutrophil extracellular trap release from PMNs also remained uninhibited by CF sputum. In summary, our data demonstrate that the CF airway environment limits killing of *S. aureus* by PMNs and provides a new in vitro experimental model to study this phenomenon and its mechanism.

## 1. Introduction

Cystic fibrosis (CF) airway disease is characterized by decreased mucociliary clearance, chronic, polymicrobial infections and robust, neutrophil-dominated inflammation. Pulmonary disease is the leading cause of morbidity and mortality in CF patients, due to the progression of chronic respiratory infections and host inflammation [1]. *Staphylococcus aureus* (*S. aureus*) is the most prevalent infectious agent in the respiratory tract of CF patients [2]. According to the United States’ Cystic Fibrosis Foundation’s annual reports in 2018 and 2019, 70% of CF patients were infected with *S. aureus*, including 25% with methicillin-resistant *S. aureus* (MRSA). *S. aureus* is one of the earliest pathogens recovered in the airways of CF children; it persists throughout the life of CF patients, and over a decade ago, surpassed *P. aeruginosa* as the most common airway pathogen in CF [3,4,5]. MRSA is associated with accelerated decline in lung function, increased hospitalization and mortality in CF [6,7]. For these reasons, it is of great importance to study *S. aureus* in CF to better understand CF lung disease [2,3].

One of the earliest abnormalities in CF airway disease is the recruitment of polymorphonuclear neutrophil granulocytes (PMNs) to the lungs. PMNs are an important component of the airway’s antimicrobial defense [7]. PMNs’ primary killing mechanisms against pathogens involve intracellular killing through phagocytosis and extracellular killing by neutrophil extracellular traps (NETs) [8,9]. PMNs represent the most important immune cell type fighting *S. aureus* including MRSA [10,11,12,13]. Antibody- or complement-enhanced phagocytosis and the associated respiratory burst generating reactive oxygen species (ROS) represent the main mechanisms by which PMNs kill *S. aureus* [10,11,14]. PMN-mediated killing of *S. aureus* in CF airways is obviously impaired since *S. aureus* is a major respiratory pathogen in CF [3,4,5]. The reason for this remains unclear, as *S. aureus* has been detected inside PMNs in CF airways indicating that phagocytosis occurs to some extent [15].

The persistence of *S. aureus* infections in the presence of PMNs in the CF lung indicates that anti-staphylococcal PMN killing mechanisms fail. We hypothesized that the CF airway environment impairs PMN-mediated clearance of *S. aureus*. The goal of our study was to determine the potential effects of the CF airway environment on antibacterial effector functions of PMNs against *S. aureus*.

## 2. Materials and Methods

### 2.1. Human Subjects

All the human subject studies were performed by following the guidelines of the World Medical Association’s Declaration of Helsinki. 

### 2.2. Control Subjects

Control human subjects recruited at the University of Georgia (UGA) provided informed consent before blood donation for PMN isolation according to the protocol UGA# 2012-10769-06. Healthy subjects were chosen to match the sex and age distributions of CF patients. While control subjects did not suffer from CF based on self-report, they could have theoretically included CFTR heterozygous subjects that are represented in the general population with an approximate 1:25 frequency and are otherwise healthy. Two healthy subjects were recruited at Emory University to donate sputum samples following induction of sputum through inhalation of a 7% hypertonic saline solution. 

### 2.3. CF Patients 

CF subjects were patients recruited at the Adult CF Clinic at Emory University. CF patients signed informed consent to provide blood and sputum samples (IRB00042577). CF diagnosis was confirmed by pilocarpine iontophoresis sweat testing and/or CFTR gene mutation analysis showing the presence of two disease causing mutations. CF participants were selected for blood draw or sputum collection only if they were clinically stable and on no new medications within the previous three weeks of the clinic visit. Sputum cultures were taken on the day of the clinic visit when the blood was drawn, and the presence or absence of *S. aureus* as identified by the clinical microbiology laboratory was noted. Baseline lung function was defined according to the guidelines of the CF Foundation Patient Registry which is the average of the best percent predicted forced expired volume in one second (FEV_1_) for each quarter of the calendar year. Blood was drawn into a silicone-coated tube and processed as above until shipped to UGA for analysis.

### 2.4. PMN and Serum Isolation

Control human subjects were recruited at UGA by the Clinical Translational and Research Unit to donate blood for PMN isolation and serum preparation. PMNs and serum were also isolated from the peripheral blood of CF patients along with age/gender-matched healthy donors at Emory University. 20–30 milliliters of blood were drawn into EDTA-coated tubes and PMNs were isolated using the EasySep™ Direct Human PMN Isolation Kit (Catalog #19666, Stem Cell technologies, Vancouver, BC, Canada) according to the manufacturer’s protocol. This protocol routinely yields 30–130 × 10^6^ live PMNs with >99.9% red blood cell depletion and ~99% purity of the isolated cells. Additional ten milliliters of blood were drawn into a separate, silicone-coated tube without anticoagulant and allowed to clot at room temperature for 30 min. The tube was centrifuged twice (1300× *g*, 5 min), the resulting supernatant (serum) was collected, and the coagulant was discarded. The serum was kept on ice for same-day experiments or frozen at −80 °C for future work.

### 2.5. Sputum Collection and Processing 

All human studies involving sputum collection from CF patients and healthy controls were approved by the Emory University Institutional Review Board and were in accordance with institutional guidelines. All donors gave consent before sputum collection. Sputum samples were processed and modified from the previously published method [16]. Briefly, samples were kept on ice at 4 °C throughout all experimental procedures. After collection, sputa were weighed, their volume measured, and 3 mLs of ice-cold PBS-EDTA (1 × PBS–5 mM EDTA) were supplemented to every 1.0 g of sputum samples. Sputum samples were repeatedly and slowly passaged through a sterile 18-gauge needle to homogenize the sputum. Cells were pelleted after low-speed centrifugation (4 °C, 800× *g*, 10 min). The sputum supernatant was transferred into microcentrifuge tubes and centrifuged (3000× *g*, 10 min, 4 °C). The clear sputum supernatants were stored in aliquots at −80 °C until analysis.

### 2.6. In vitro “CF Sputum Model”

To test whether the CF airway environment alters PMN effector functions, an in vitro model was established using blood PMNs isolated from healthy donors and exposed to CF sputum supernatants. Sputum supernatants were pooled from three individual CF patients and called “sputum cocktails”. Five different sputum cocktails were tested. Healthy blood PMNs (10^7^ /mL) were pretreated with 30% sputum cocktail for 3.5 h at 37 °C resulting in a final concentration of 1.5 mM EDTA in the sputum. At the end of incubation, PMNs were washed twice with the assay medium consisting of 1 × HBSS, 10 mM HEPES, 5 mM glucose, and 1% (*v/v*) autologous serum to remove extracellular sputum components and to prevent their direct interaction with subsequently added bacteria. Additional control experiments showed that these two washes were sufficient to avoid a potential direct contact of any, minimal, residual, carryover CF sputum and bacteria. PMNs used as control samples not exposed to CF sputum were kept in undiluted assay medium without CF sputum for the same amount of time.

### 2.7. Bacteria

Eight *S. aureus* isolates recovered from the respiratory tract culture of CF patients were obtained from the Emory Cystic Fibrosis Biospecimen Repository (CFBR). Four MRSA and four MSSA isolates were used in this work. The age of the four CF patients donating the MSSA strains ranged between 4 and 22 years while those of the MRSA donors ranged between 20–53 years. The antibiotic sensitivity profile of the isolates has been determined. USA300, was used as a laboratory reference strain [17,18].

The *S. aureus* strain JE2 expressing red fluorescence protein (SA-rfp) was constructed by isolating pHC48 from RN4220 [19,20], a restriction-deficient *S. aureus* strain, and electroporating it into JE2. Briefly, electrocompetent cells of JE2 were made by using a modified procedure from Lofblom et al. [21]. The restriction system was subsequently inactivated at 56 °C and bacteria were resuspended in 10% glycerol + 500 mM sucrose. Finally, the pHC48 plasmid was electroporated using a pulse at 2.1 kV and cells were recovered in TSB + 500 mM sucrose before plating on TSA with 10 μg/mL chloramphenicol to select for cells that had successfully accepted pHC48. All the clinical isolates and strains of *S. aureus* used in this work are listed in Table 1.

For all the experiments, *S. aureus* was grown on blood agar (TSA II 5% SB) at 37 °C. The following day, bacteria were cultured from the agar plates in optimal growth medium (2 mL LB medium) at 37 °C shaking for 2–3 h. The bacteria were harvested and centrifuged at 10,000× *g* for 5 min, supernatants were discarded, and the bacterial pellets were washed twice with 1 × HBSS. After the last wash, the bacterial pellets were resuspended in 1mL 1 × HBSS and optical density was measured in a 96-well microplate at 600 nm in Varioskan flash™ microplate spectrophotometer (Thermo Fisher Scientific, Waltham, MA, USA). Based on previous experience, bacterial optical density values of 0.6 were determined to correspond to 1.0 × 10^9^/mL bacterial concentration [23]. Calculated bacterial concentration values were confirmed in every experiment by colony counting. The bacteria were then opsonized with 10% (*v/v*) autologous serum of the PMN donor (healthy control or CF patient) for 20 min at 37 °C. Following opsonization, the bacteria were washed by centrifugation at 10,000× *g* for 5 min and resuspended in assay medium.

### 2.8. Cell Viability 

Human PMNs were incubated for 3.5 h in 1.5 mL microcentrifuge tubes at a concentration of 1 × 10^6^ cells/100 µL assay medium (HBSS + 1% autologous serum + 5 mM glucose + 10 mM HEPES) with or without 30% of the CF sputum cocktail. Following incubation, cells were washed and resuspended in 1X PBS. Cell were then stained with a Zombie Aqua Fixable Viability Kit (Biolegend, San Diego, CA, USA; cat# 423102) at a dilution of 1:10,000 for 15 min at room temperature, protected from light. Cells were collected by centrifugation and washed with 1 × PBS containing 1% BSA. PMN viability was also assessed by flow cytometry in some experiments. The granulocyte marker CD66b conjugated to PerCP-Cy5.5 fluorochrome (Biolegend, San Diego, CA, USA; cat# 305108) was used to identify PMNs in the cell suspension. Cells were suspended in 500 µL BD Stabilizing Fixative (BD) and analyzed at the University of Georgia College of Veterinary Medicine Cytometry Core Facility on an LSRII Flow Cytometer (BD, San Jose, CA, USA), using the violet laser the 525/50 filter for Zombie Aqua detection, and the 488 nm blue laser with the 695/40 filter for PerCP/Cy5.5 detection. Data were analyzed with BD FACsDiva™ software (BD Biosciences, San Jose, CA, USA).

### 2.9. High Throughput Bacterial Killing Assay

To assess bacterial killing, a high throughput 384-well microplate-based assay was used that is the improved and expanded version of our previously described, 96-well microplate-based protocol to assess bacterial survival [24]. Briefly, purified human PMNs and opsonized bacteria were mixed at a ratio of 10:1 multiplicity of infection (MOI, *S. aureus*: PMN)) in microcentrifuge tubes. The tubes were incubated and mixed regularly for 30 min at 37 °C to ensure appropriate mixing of bacteria and PMNs. For assessing the killing of bacteria by PMNs treated with sputum, untreated PMNs were used as control. After 30 min, PMNs were lysed with 1 mg/mL saponin in 1 × HBSS (on ice for 5 min). Samples were then diluted in 1 × HBSS 100-fold in a separate microcentrifuge tube on ice. The 384-well microplate was placed on ice and 80 µL of LB broth was placed in each well. 20 µL volumes of each experimental sample were pipetted into corresponding wells in quadruplicates. As a negative control, saponin dissolved in corresponding HBSS/LB medium mix was placed in wells to ensure detection of potential external bacterial contamination. For absolute quantitation, a standard curve was established with known bacterial concentrations for each bacterial strain or isolate tested that were applied in every experiment. The standard curves of each CF isolate were highly reproducible between experiments. To determine the starting bacterial inoculum (0 min), bacteria were added to PMNs and immediately (few seconds later) lysed with saponin. In our experience, this is a more appropriate time zero control than using only bacteria because the sample also includes PMNs. Once all samples were added to the microplate, the plate was placed in the incubator at 37 °C for 15 min to warm up evenly and then placed in a microplate spectrophotometer Varioskan Flash (Thermo Scientific, Waltham, MO, USA). The microplate reader measured bacterial growth in each well at 600 nm absorbance every 4 min for 16 h with constant heating at 37 °C and 5 s of shaking prior to each read to prevent bacterial settlement. After the measurement, growth curves were generated, the initial bacterial concentrations in each sample were determined and bacterial killing efficiencies were assessed and expressed as decrease in surviving bacteria over time [24].

### 2.10. Attachment and Phagocytosis

Attachment/binding and phagocytosis of *S. aureus* to PMNs was assessed by flow cytometry. Bacterial attachment to PMNs was quantified using the *S. aureus* strain JE-2 expressing red fluorescence protein (SA-rfp). Healthy human PMNs were isolated and incubated with or without 30% sputum supernatant as previously described. PMNs were then washed twice to remove the sputum and were resuspended in assay medium. Since infecting PMNs with SA-rfp alone will not be able to distinguish between *S. aureus* being attached to the outside of the cells or taken up inside the phagosome, human PMNs were pretreated with the cytoskeleton inhibitor, cytochalasin-B (10 µM, 30 min at 37 °C), to prevent phagocytic uptake of *S. aureus*. SA-rfp was opsonized with 10% autologous serum of the PMN donor and added to PMNs at MOI of 10. To assess attachment/binding to PMNs, SA-rfp/PMN co-cultures were incubated with frequent mixing for 30 min at 37 °C, then washed twice with cold 1 × PBS and resuspended in PBS. Cells were stained with a Zombie Aqua™ Fixable Viability Kit (Biolegend, San Diego, CA, USA, cat#423102) at a dilution of 1:5000 for 15 min at room temperature, protected from light. PMNs were then washed and resuspended in 1 × PBS containing 1% BSA and stained with the PMN marker CD66b conjugated to PercP-Cy5.5 fluorochrome (Biolegend, cat#305108) at a final concentration of 1 µg/mL for 30 min, protected from light. PMNs were washed and resuspended in BD stabilizing Fixative and analyzed by the NovoCyte Quanteon 4025. For zombie aqua detection, the violet laser at 405 nm with 525/50 filter was used, the blue laser at 488nm with the 695/40 filter was used for PerCP/Cy5.5 detection, and the yellow laser at 561nm with the 586/20 filter was used for rfp.

Phagocytosis was measured in PMNs that were purified and treated with the CF sputum as described above. The CF isolate MRSA24 was stained with 5 mM pHrodo™ iFL green STP Ester (Thermofisher, cat#P36012) for 1 h at 37 °C, protected from light. After the staining MRSA24 was opsonized by the addition of 10% autologous serum of the PMN donor and added to PMNs at MOI of 10. MRSA24/PMN co-cultures were incubated for 1 h at 37 °C protected from light with consistent mixing. PMNs were then washed with cold 1xPBS twice and resuspended in PBS to be stained with a Zombie Red™ Fixability Kit (Biolegend, cat#423109) at a dilution of 1:5000 for 15 min at room temperature. PMNs were then washed and resuspended in eBioscience™ flow cytometry staining buffer (Thermo Fisher Scientific, Waltham, MA, USA, cat#00-4222-26). PMNs were then stained with the granulocyte marker CD66b AlexaFlour 647 (Biolegend, cat#561645) at a final concentration of 1 μg/mL for 30 min, protected from light. PMNs were washed and resuspended in BD stabilizing fixative. For zombie red detection, the yellow laser at 561 nm was used with the 615/20 filter, the red laser at 637 nm with the 660/20 laser was used for CD66B AlexaFluor 647detection, and the blue laser at 488 nm with the 530/30 laser was used for pHrodo green detection.

To assess attachment and phagocytosis of *S. aureus*, all the data were analyzed at the University of Georgia College of Veterinary Medicine Cytometry Core Facility on a NovoCyte Quanteon 4025 with NovoSamplerQ utilizing NovoExpress software v.1.4.1, Agilent, Santa Clara, CA, USA.

### 2.11. NADPH Oxidase Activity Measurements

ROS production was measured using the Diogenes-based chemiluminescence kit (National Diagnostics, Atlanta, GA) as before [23,25,26,27]. Shortly, 250,000 PMNs were allowed to adhere to 96-well solid white plates for 1 h at 37 °C in assay medium (previously described). Cells were stimulated by *S. aureus* isolates (10 MOI), PMA (100 nM) or left unstimulated. Chemiluminescence was measured by a Varioskan Flash microplate luminometer (Thermo Scientific, Waltham, MA, USA) for 90 min. ROS production data are shown as kinetics of representative curves (relative luminescence units, RLU) or integrated superoxide production by analyzing accumulated luminescence for the entire (60 min) or partial (15 min) duration of the measurement and normalizing it on the PMA-stimulated signal as 100%. Superoxide generation of PMNs was specifically tested by the superoxide dismutase-inhibitable cytochrome-c reduction assay as described earlier [24,28]. To measure extracellular superoxide production, PMNs were suspended in assay medium containing 50 μM of cytochrome-c (Sigma, cat#C3131). The cell suspension was added into a 96-well plate and incubated at 37 °C for 5 min in a shaking microplate spectrophotometer Varioskan Flash (ThermoScientific). PMNs were activated with 100 nM PMA, indicated *S. aureus* CF clinical isolates or Zymosan A particles from *Saccharomyces cerevisiae* (Sigma, cat#Z4250) opsonized in 10% of autologous serum of the PMN donor at MOI of 10. The increases in absorption at 550 nm were recorded for 60 min with two measurements/min at 37 °C. Superoxide production was calculated with the use of an absorption coefficient of 21 mM^−1^ cm^−1^ for cytochrome-c according to the Lambert-Beer law and expressed as nmol O_2_^.−^/10^6^ PMNs/hr.

### 2.12. NET Release

DNA release from human PMNs was quantitated as described [29]. Briefly, 250,000 PMNs/well were seeded on 96-well black transparent bottom plates and incubated for 30 min at 37 °C. Next, 0.2% Sytox Orange (Life Technologies, Grand Island, NY, USA) membrane-impermeable DNA-binding dye was added to PMNs and PMNs were infected with 10:1 MOI of the indicated *S. aureus* isolates. Fluorescence (excitation: 530 nm, emission: 590 nm) was recorded for up to 8 h in a fluorescence microplate reader (Varioskan Flash, Thermo Fisher Scientific, Waltham, MA, USA) at 37 °C. Relative fluorescence unit (RFU) results were normalized on the signal obtained in PMA-stimulated PMNs and expressed as its percentages. Unstimulated PMNs were used as the negative control.

Citrullination of PMN histone H3 protein was also used as a measure of NET formation. Purified PMNs were incubated for 3.5 h with or without 30% CF sputum supernatants, as described above. Following the incubation, PMNs were washed twice with 1x PBS to remove the sputum and resuspended in PBS. Cells were stained with a Zombie Aqua™ Fixable Viability Kit (Biolegend, cat#423102) at a dilution of 1:5000 for 15 min are room temperature, protected from light. PMNs were washed with PBS/1% BSA, and then fixed and permeabilized with the Fix and Perm/ Cell Fixation and Permeabilization Kit (Abcam, Cambridge, MA, USA) following manufacturer’s instructions. All subsequent cell processing was performed on ice, protected from light. The primary anti-histone H3 (citrulline R2 + R8 + R17) (Abcam, Cambridge, MA, USA) antibody was incubated with the cells for 30 min. Cells were washed with PBS/ 1% BSA followed by incubation with goat-anti-rabbit-FITC (BD PharmingenTM, Franklin Lakes, NJ, cat#554020) for 30 min. Cells were washed with PBS/ 1% BSA and stained with the granulocyte marker CD66b conjugated to PercP-Cy5.5 fluorochrome (Biolegend, cat#305108) for 30 min. PMNs were washed with PBS/1% BSA and resuspended in 500 µL Stabilizing Fixative (BD Biosciences, San Jose, CA, USA), and stored at 4 °C until analysis. Samples were read at the University of Georgia College of Veterinary Medicine Cytometry Core Facility on a BD LSRII flow cytometer (BD Biosciences, San Jose, CA, USA) within 24 h of staining. Data were analyzed with the BD FACsDiva™ software (BD Biosciences, San Jose, CA, USA).

### 2.13. DNAse Activity Measurement

DNAse activity was measured by a fluorometric assay kit (BioVision, Milpitas, CA, USA; cat #: K429-100) detecting DNAse enzyme activity by cleavage of a DNA probe to yield a fluorescent product. The *S. aureus* isolates were prepared in molecular biology-grade water using filter tips to prevent DNA contamination. A DNA probe standard curve was generated following the protocol ranging from 0–20 pmol/well. 50 µL of the DNA probe standards and of the bacterial samples were added to a white, 96-well plate in triplicates and 50 µL of the reaction mix were added directly after. Fluorescence was measured in kinetic mode every 30 s for 90 min at 37 °C. RFU values were applied at each time point to the standard curve equation generated to determine pmol of DNA cleaved at each reaction point (pmol/min).

To confirm results produced by the DNAse I fluorometric assay, Remel™ DNASE test agar was also used (Thermo Fisher Scientific, cat #: R453252). The test agar was suspended in demineralized water and autoclaved for sterility. *S. aureus* isolates were grown overnight to generate single colonies. Three colonies from each isolate were taken and smeared in a single-straight line in the middle of the agar dish (one isolate per Petri dish). The test agar dishes were incubated overnight at 37 °C. The next day, the test agar with grown *S. aureus* cultures were flooded with 1N HCL for 5 min. The 1N HCL was aspirated and the DNA clearing zones were measured with a ruler in mm.

### 2.14. Apoptosis

Apoptosis of human PMNs was measured by flow cytometry using the Apotracker(TM) Green (Biolegend, San Diego, CA; cat# 423102) apoptosis probe in combination with propidium iodide (Sigma Aldrich, St. Louis, MO, USA) staining. Cells were stained with Apotracker(TM) Green following manufacturer’s protocol, protected from light. PMNs were collected by centrifugation, washed twice with 1X PBS containing 1% BSA, resuspended in 300 uL 1 × PBS containing 1% BSA. Propidium iodide was first diluted at a 1:1 ratio in 2 × PBS and then added to the cells at a 1:10 dilution. Samples were immediately analyzed at the UGA Veterinary Medicine Cytometry Core Facility on a NovoCyte Quanteon 4025 with NovoSamplerQ utilizing NovoExpress software v.1.4.1 (Agilent, Santa Clara, CA, USA).

### 2.15. Statistical Analysis

Results of multiple bacterial isolates obtained by the high-throughput killing assay were analyzed by one-way ANOVA and Tukey’s multiple comparison test. Results between PMNs treated with sputum and no sputum were analyzed by two-tailed, paired Student’s t-test. Results between two patient cohorts were analyzed by Mann–Whitney test. Correlation between two parameters was evaluated with Spearman’s rank-order correlation. Data are expressed as mean plus-minus standard error of mean (SEM). The correlation coefficient (r) was calculated. Statistically significant differences were considered as *, *p* < 0.05; **, *p* < 0.01; ***, *p* < 0.001. Statistical analysis was carried out with GraphPad Prism version 6.07 for Windows software (GraphPad Software, San Diego, CA, USA).

## 3. Results

### 3.1. In vitro Model of CF Airway-Like Conditions 

To investigate whether the CF airway environment affects PMNs’ ability to kill bacterial pathogens, we established a new in vitro experimental model. In this model, human PMNs isolated from the blood of healthy volunteers are exposed to pooled supernatants of sputum samples collected from CF patients to mimic the CF airway environment. This treatment referred to as “CF sputum model” throughout the manuscript has been optimized and involves a 3.5 h-incubation of human PMNs with 30% (*v/v*% diluted in assay medium) CF sputum supernatant pooled from three independent CF patients in equal proportions. 

### 3.2. CF Sputum Exposure Does Not Impair PMN Viability 

**Figure 1:**
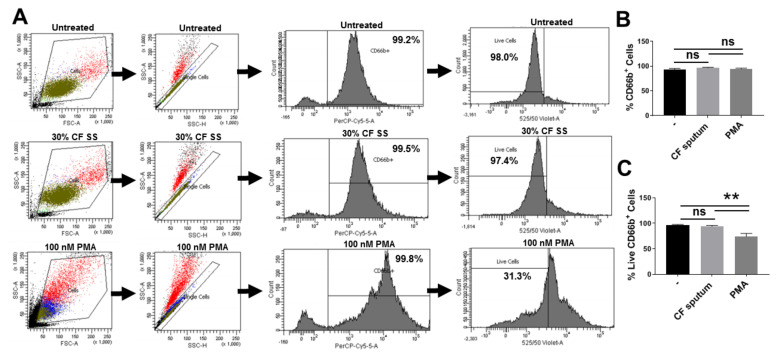
CF sputum supernatant treatment does not affect PMN viability. PMN purity and viability following isolation from blood and subsequent 3.5 h-CF sputum incubation was measured by flow cytometry using the Zombie Aqua Cell Viability Kit™. (**A**) Representative images of the gating strategy used to determine the percent of viable PMNs (CD66b^+^/Zombie Aqua^−^) for each condition tested are shown. (**B**) Flow cytometric analysis showed no significant difference in the percent of PMNs and CD66 surface expression among the conditions tested (*n* = 15). (**C**) CF sputum treatment does not impair PMN viability under the conditions used in this study. Treatment with 100 nM PMA for 30 min, however, results in a significant decrease in PMN viability compared to both the sputum supernatant-treated and untreated PMNs (*n* = 15). One-way ANOVA, Tukey’s multiple comparison test. **, *p* < 0.01; ns, not significant. PMA, phorbol 12-myristate 13-acetate; CF SS; Cystic fibrosis sputum supernatant.

It is relevant to confirm that the CF sputum model does not affect the viability of PMNs. Viability was defined as maintenance of membrane barrier function of PMNs that is a widely accepted measure in any cell. Viability was measured based on the general principle of active dye extrusion of living cells using the Zombie Aqua dye. A representative of the flow cytometry gating strategy used to assess the viable percentage (Zombie Aqua-negative) of purified PMNs (CD66b-positive cells) is shown in Figure 1A. The sputum treatment does not influence the surface expression of the PMN marker CD66b (Figure 1B). There was no significant difference in cell viability between healthy blood PMNs exposed to the CF sputum cocktail and those incubated in assay medium only. In contrast, PMNs treated with 100 nM PMA for 30 min showed a significant and expected reduction in viability to an average of 74% when compared to sputum-supernatant treated or untreated PMNs (Figure 1C). These results confirm that the CF sputum exposure does not impact the viability of human PMNs and subsequent observations do not result from PMN plasma membrane damage.

### 3.3. CF Sputum Does Not Induce Apoptosis in Human PMNs

**Figure 2:**
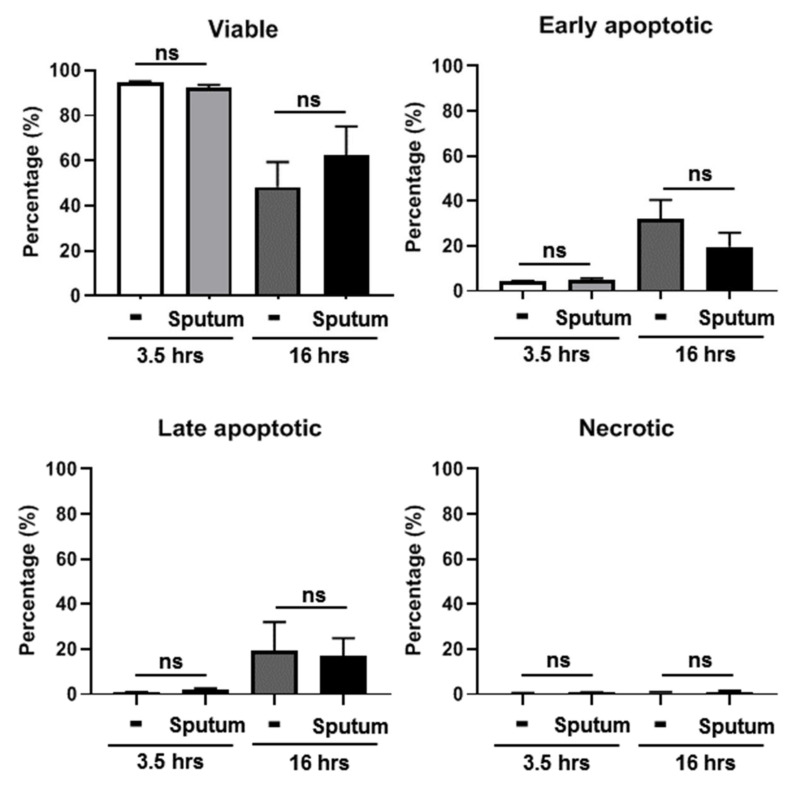
CF sputum supernatant does not induce apoptosis or necrosis in human PMNs. Human blood PMNs were incubated in CF sputum supernatants for 3.5 or 16 h prior to fluorescence staining with Apotracker (TM) Green apoptosis probe and propidium iodide (PI) viability dye. The following cell populations were identified by flow cytometry: viable (double negative), early apoptotic (PI-negative, Apotracker-positive), necrotic (PI-positive, Apotracker-negative) and late apoptotic (double positive). Results are expressed as mean ± S.E.M (*n* = 6). One-way ANOVA, Dunn’s multiple comparison test. ns, not significant.

The exposure of human PMNs to the CF sputum supernatant could also lead to the induction of apoptosis. To address this, human PMNs were exposed to CF sputum for 3.5 and 16 h and apoptosis was assessed by flow cytometry to identify the following cell populations: viable, early apoptotic, necrotic and late apoptotic. The CF sputum treatment did not increase the proportion of early or late apoptotic cells (Figure 2). On the contrary, there was a trend, although not significant, towards the CF sputum delaying spontaneous apoptosis and increasing the proportion of viable PMNs after 16 h of ex vivo incubation (Figure 2). CF sputum did not cause PMN necrosis even after 16 h of incubation (Figure 2). These results confirm that the CF sputum does not lead to necrotic or apoptotic death of PMNs.

### 3.4. CF Isolates of S. aureus Are Killed by Human PMNs

Next our goal was to explore the effect of CF sputum treatment on PMNs’ ability to kill CF respiratory pathogens. Table 1 displays the *S. aureus* CF clinical isolates from 8 different CF subjects that were used in this study. Four MRSA isolates and four MSSA isolates obtained from CF patients were selected from Emory’s CFBR. Whole-genome sequences are available with the assembly metrics for all *S. aureus* isolates [30].

**Figure 3:**
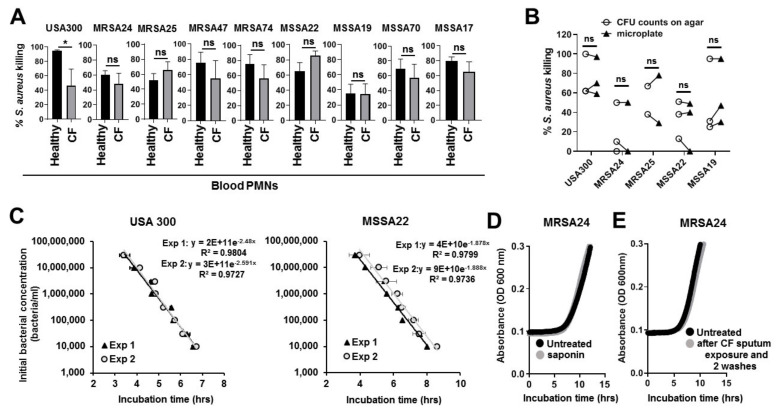
Healthy and CF human blood neutrophils kill CF isolates of *S. aureus* in vitro. (**A**) Healthy and CF human blood PMNs were infected with the *S. aureus* reference strain (USA300), 4 MRSA isolates, and 4 MSSA isolates collected from CF patients. All isolates were infected at MOI of 10 except MSSA70 and MSSA17 that were infected at MOI of 5. Bacterial killing was measured by the high-throughput microplate-based assay. Mean ± S.E.M, *n* = 6. Two-tailed, paired Students’ *t*-test. (**B**) Comparison of PMN-mediated *S. aureus* killing by two methods: high-throughput microplate-based assay and agar plate based colony counting assay. Lines connect data derived from the same, individual experiment and same samples via the two different methods. Experiments were repeated three times on three independent human donors’ neutrophils. (**C**) Standard curves of USA 300 and MSSA22 growth calibrations are shown as examples indicating the tight correlation between initial bacterial concentrations (*y* axis) and the incubation time values (*x* axis). Two representative curves for each strain are shown in the same graph with trend lines, the equation and R^2^ values. Mean ± S.D. (**D**) Representative growth curves of MRSA24 with or without saponin treatment that is used in the killing assay (*n* = 3). (**E**) Representative growth curves of MRSA24 without any treatment or in medium that was used to resuspend human PMNs after CF sputum exposure for 3.5 h and two washes according to the protocol of the killing assay. These data show that two washes of human PMNS are sufficient to ensure that the CF sputum exposure of PMNs does not interfere with subsequent growth of tested bacteria. CFU, colony-forming unit; ns, not significant; OD, optical density.

Killing of *S. aureus* CF clinical isolates by healthy PMNs was measured by the 384-well microplate-based killing assay [24]. USA300 was used as a laboratory reference strain (Table 1) [17,18]. Our result show that all CF isolates of *S. aureus* could be killed by healthy PMNs (Figure 3A). To explore whether CFTR deficiency of human blood PMNs would affect their ability to kill *S. aureus*, the eight *S. aureus* CF isolates previously studied were also subjected to measure their killing by CF PMNs using the microplate-based killing assay. As results in Figure 3A show there were no significant differences in PMN-mediated MRSA or MSSA clearance between non-CF and CF PMNs. Interestingly, only killing of the laboratory strain USA 300 was significantly impaired in CF PMNs compared to healthy cells (Figure 3A). Therefore, we conclude that human normal and CF blood PMNs are competent in killing *S. aureus*.

**Figure 4:**
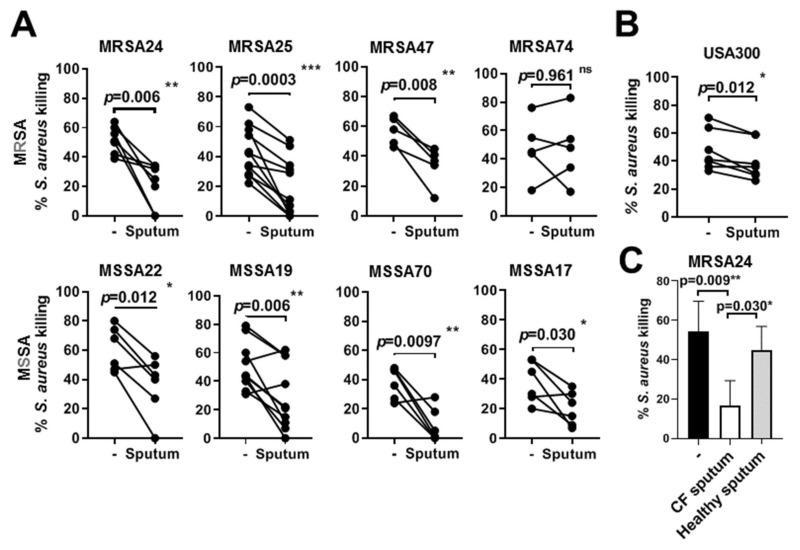
CF sputum treatment impairs neutrophils’ ability to kill *S. aureus*. PMNs were first treated with 30% (*v/v*) CF sputum cocktail and then infected with the *S. aureus* lab strain (USA300), 4 MRSA isolates and 4 MSSA isolates collected from CF patients. Bacterial killing was measured by a high-throughput microplate-based assay. (**A**) The effect of CF sputum pretreatment on PMN-mediated killing of *S. aureus* CF clinical isolates (*n* = 5–11) or (**B**) USA 300 (*n* = 7) is shown. All isolates were infected at MOI of 10 except MSSA70 and MSSA17 were infected at MOI of 5. Mean ± S.E.M. Data were analyzed by Wilcoxon matched-pairs signed rank test. (**C**) The effect of pretreatment with sputum cocktails isolated and pooled from CF patients or healthy controls (*n* = 2) on PMN-mediated killing of MRSA24 (*n* = 4). Mean ± S.E.M. Two-tailed, paired Students’ *t*-test (**A**,**B**) or one-way ANOVA test (**C**) were used. Statistically significant differences were considered as *, *p* < 0.05; **, *p* < 0.01; ***, *p* < 0.001. Ns, not significant.

To further confirm this with classical methodology, the high-throughput killing assay results were repeated by agar plate-based colony counting assays in case of four CF isolates (two randomly picked MRSA, and 2 MSSA isolates) (Figure 3B). Results generated with both methods reveal the same extent of killing with no significant differences between the results. To further characterize the microplate-based killing assay, we show representative data for USA300 and MSSA22 indicating a tight correlation between initial bacterial concentrations and incubation time values (Figure 3C). Moreover, additional control experiments confirm that the use of saponin under the conditions of the killing assay does not interfere with *S. aureus* growth (Figure 3D). To exclude the possibility that some residual sputum will be left with PMNs (despite extensive washes) that could affect *S. aureus*, we exposed human PMNs to CF sputum for 3.5 h, washed the cells subsequently twice with PBS according to the protocol and collected the supernatants of the last wash buffer. As shown in Figure 4E, this wash buffer did not have any inhibitory action on the exponential growth of *S. aureus* MRSA24. Thus, the CF sputum treatment or steps of the killing assay protocol do not interfere with *S. aureus* growth further confirming that the 384-well plate-based, high-throughput killing assay described here represents an efficient and reliable way to measure *S. aureus* killing, equivalent to the classical colony-counting method.

### 3.5. CF Sputum Compromises the Killing of S. aureus Clinical Isolates by PMNs 

To determine whether the CF sputum inhibits PMN-mediated *S. aureus* killing, PMNs from healthy donors were pretreated with CF sputum supernatant and bacterial killing was measured using all eight *S. aureus* CF isolates simultaneously via the microplate-based killing assay. Each trend line connecting the points from ‘no sputum’ to ‘sputum’ represents one experiment. When occasionally the bacteria were not killed more than 20% by healthy PMNs (in the absence of sputum treatment), the data were excluded as a strong enough baseline of bacterial killing by PMNs was not achieved. Seven out of the eight CF *S. aureus* isolates tested showed a significant reduction in PMN-mediated killing upon sputum treatment (Figure 4A). PMNs were infected with a MOI of 5 with MSSA70 and MSSA17, as both isolates were highly resistant to killing at MOI of 10. Interestingly, clearance of only one *S. aureus* isolate, MRSA74, by PMNs was not compromised by the sputum treatment (Figure 4A). Killing of the *S. aureus* control strain, USA 300, was significantly inhibited by the CF sputum (Figure 4B). To address whether the observed inhibitory effect of the CF sputum is unique to CF, we also tested sputa collected from two healthy controls. As the results in Figure 4C show, killing of the tested *S. aureus* CF isolate (MRSA24) was only inhibited by CF sputum, not by sputa from control subjects. Altogether, we provide evidence to show that CF sputum inhibits the killing of *S. aureus* clinical isolates by PMNs.

### 3.6. CF Sputum Does Not Inhibit PMN Attachment and Phagocytosis of S. aureus

**Figure 5:**
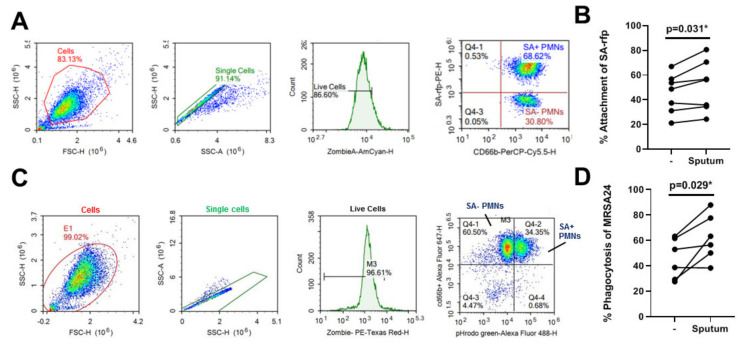
CF sputum treatment does not impair *S. aureus* binding and phagocytosis by PMNs. PMNs were isolated from healthy donors and exposed to CF sputum supernatant. To measure bacterial attachment, PMNs were infected with fluorescently labeled, opsonized *S. aureus* (SA-rfp) (10 MOI). (**A**) Representative images of the gating strategy used to determine the percent of SA-rfp attached PMNs (CD66b^+^/Zombie Aqua^-^) for each condition. (**B**) Comparison of the attachment of SA-rfp to PMNs untreated or treated with CF sputum supernatant (*n* = 7). (**C**) To determine phagocytosis, MRSA24 CF isolate was labelled with the pH-sensitive dye pHrodo, opsonized and exposed to PMNs. Representative images of the gating strategy used to determine the percent of MRSA24 phagocytosed by PMNs for each condition. (**D**) Comparison of MRSA24 phagocytosis by PMNs that were untreated or treated with CF sputum supernatant (*n* = 6). Two-tailed, paired Students’ *t*-test. *, *p* < 0.05.

Phagocytosis is the main mechanism by which PMNs kill *S. aureus*. Phagocytosis involves two steps, bacterial attachment to the PMN surface that is followed by uptake into the phagosome. To determine whether the first step, bacterial binding to PMNs, is affected by the CF sputum, flow cytometry was utilized. A *S. aureus* strain JE-2 expressing red-fluorescence protein (SA-rfp) was generated and used to quantify the binding of *S. aureus* by PMNs in presence of cytochalasin B to inhibit bacterial uptake. A representative of the flow gating strategy used to assess the percent of *S. aureus* attached to live PMNs (CD66b-positive, zombie-negative cells) is shown in Figure 5A. The same number of PMNs were tested in each condition. Our results found that there was no impairment of *S. aureus* binding to PMNs by the CF sputum (Figure 5B). On the contrary, a small but significant increase in binding of *S. aureus* to PMNs was observed (Figure 5B). Phagocytosis was quantified using flow cytometry by staining the CF isolate MRSA24 with the pH-sensitive dye pHrodo to detect bacteria only inside the phagosome. A representative flow gating strategy used to assess the percent of *S. aureus* inside phagosomes of purified, live PMNs (CD66b-positive, zombie-negative cells) is shown in Figure 5C. Similar to attachment, there is enhanced phagocytosis of *S. aureus* by PMNs incubated with CF sputum compared to those incubated with assay medium alone (Figure 5D). Altogether, our data show that the CF sputum supernatant does not inhibit *S. aureus* binding to or phagocytosis by PMNs. 

### 3.7. CF Sputum Does Not Inhibit PMN Superoxide Production in Response to S. aureus 

**Figure 6:**
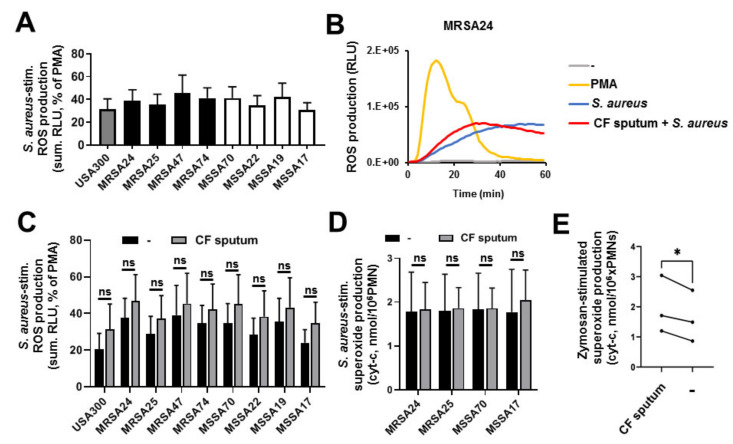
CF sputum does not impair PMN superoxide production in response to CF isolates of *S. aureus*. Human blood PMNs were exposed to the indicated isolates of *S. aureus* (10 MOI) and ROS production was measured by Diogenes-based chemiluminescence (**A**–**C**) or cytochrome-c reduction assay (**D**–**E**). (**A**) PMNs respond to the presence of CF bacterial isolates with robust ROS production (mean ± S.E.M, *n* = 6–7). (**B**) Representative kinetics of *S. aureus*-stimulated PMN ROS release curves (60 min) (*n* = 7). In total, 100 nM PMA was used as a positive control. The effect of CF sputum exposure on (**C**) ROS production or (**D**) extracellular superoxide generation in human PMNs exposed to the indicated isolates of *S. aureus* is shown. ROS production was calculated for 60 min (*n* = 6–7) by Diogenes-based chemiluminescence while superoxide production was measured for 60 min by the cytochrome-c reduction assay (*n* = 3). Mean ± S.E.M. Data were analyzed by Wilcoxon matched-pairs signed rank test. (**E**) Comparison of superoxide production by sputum-treated PMNs vs. sputum-untreated cells following exposure to zymosan (10 MOI, opsonized). Two-tailed, paired Students’ *t*-test. Statistically significant differences were considered as *, *p* < 0.05. Ns, not significant.

Oxidative killing mechanisms of human PMNs are essential to kill S. aureus, both in vitro [24] and in vivo [31]. We therefore hypothesized that the CF sputum decreases ROS production in PMNs stimulated with CF isolates of *S. aureus*. ROS generation by the PMN NADPH oxidase was stimulated by each of the *S. aureus* isolates used in the study (Figure 6A). *S. aureus* exposure stimulated an ROS signal that was comparable to that obtained by the positive control PMA (Figure 6B). No substantial differences could be observed between CF sputum-treated and untreated PMNs after *S. aureus* stimulation (Figure 6C). To confirm these results with a classical detection method specific to superoxide anions, the superoxide dismutase-inhibitable cytochrome-c reduction assay was used. These results further confirmed that there is no significant difference in the superoxide output between CF sputum-treated PMNs vs. untreated PMNs after infection by *S. aureus* (Figure 6D). No inhibition of superoxide production was observed either when a nonliving particle, zymosan, was used in PMNs (Figure 6E). Overall, these results indicate that CF sputum pretreatment does not impair PMNs’ ability to generate ROS in response to S. aureus.

### 3.8. CF Sputum Does Not Inhibit NET Release of PMNs in Response to S. aureus

**Figure 7:**
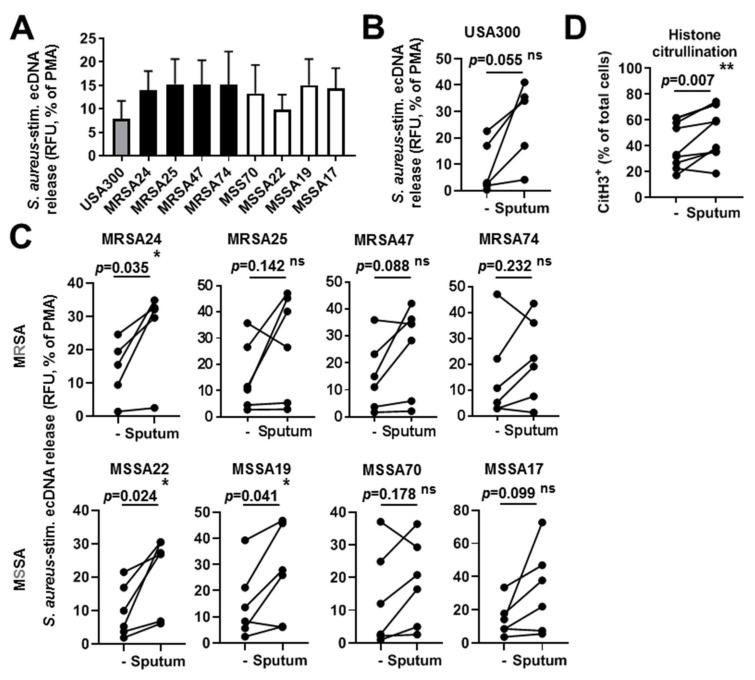
NET formation triggered by CF isolates of *S. aureus* is not compromised by CF sputum. Human blood PMNs were exposed to the indicated isolates of *S. aureus* (10 MOI) and extracellular DNA (ecDNA) release was measured for up to 8 h in presence of the membrane-impermeable, DNA-sensitive fluorescent dye, Sytox Orange. (**A**) EcDNA release in *S. aureus*-stimulated PMNs after 8 h measured as increase in fluorescence. The signal by unstimulated PMNs was subtracted and the *S. aureus*-induced ecDNA signal was normalized on the signal obtained by PMA stimulation (100 nM). Mean ± S.E.M, *n* = 5–6. (**B**) The effect of CF sputum treatment on ecDNA release in PMNs exposed to USA300. Mean ± S.E.M, *n* = 6. (**C**) The effect of CF sputum treatment on ecDNA release in PMNs exposed to the indicated CF isolates of *S. aureus*. Mean ± S.E.M, *n* = 6. (**D**) Histone H3 citrullination in PMNs exposed to the CF sputum in the absence of bacterial stimulation measured by flow cytometry (*n* = 9). Two-tailed, paired Students’ *t*-test. Statistically significant differences were considered as *, *p* < 0.05; **, *p* < 0.01. Ns, not significant.

Neutrophil extracellular traps (NET) represent an extracellular, antimicrobial trapping and killing mechanism of PMNs [9]. NETs are released from PMNs in presence of *S. aureus* and have been detected in CF airways [9,32]. Therefore, we hypothesized that NET formation of PMNs induced by *S. aureus* CF isolates is also detectable in our experimental system. We also aimed at exploring whether *S. aureus*-stimulated NET extrusion is affected by the CF sputum treatment. PMNs released NETs in response to all *S. aureus* isolates tested (Figure 7A). CF sputum pretreatment of PMNs did not lead to decreased NET release in the case of any of the bacteria (Figure 7B,C). Consistently, each line represents one experiment with a different healthy PMN donor. On the contrary, sputum exposure resulted in significantly enhanced NET release in case of three of the eight CF *S. aureus* isolates (Figure 7C). This was further confirmed by an independent method, measuring histone H3 citrullination by flow cytometry on uninfected PMNs treated with the CF sputum cocktail (Figure 7D). Overall, NET formation was not inhibited by CF sputum treatment in PMNs stimulated with *S. aureus*. 

### 3.9. S. aureus CF Clinical Isolates Possess DNAse I Activity

**Figure 8:**
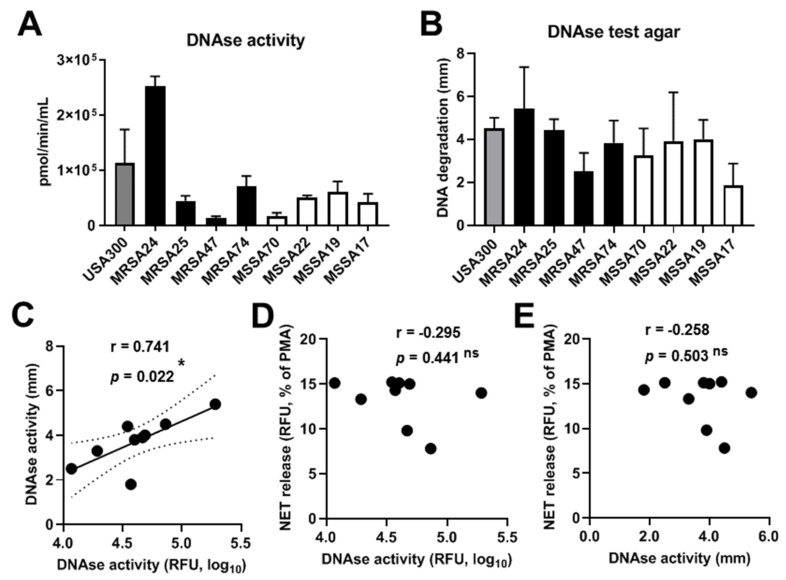
DNAse activity of CF clinical isolates of *S. aureus*. DNAse activity was measured in the indicated CF isolates of *S. aureus* by (**A**) a fluorescent enzymatic activity assay (*n* = 3–6), or (**B**) a DNAse I agar plate-based assay (*n* = 3). Mean ± S.E.M. Correlation analysis is shown between (**C**) the two DNAse activity measures, (**D**) NET release and results of the fluorescent DNAse activity assay, and ^®^ NET release and results of the plate-based DNAse activity assay. Pearson’s correlation coefficient(r). Statistically significant differences were considered as *, *p* < 0.05. Ns, not significant.

Since we observed two types of responses among the CF isolates with regard to their NET release (NET formation was either enhanced or unaffected by CF sputum), and long-term survival of *S. aureus* in CF airways has been associated with its DNAse activity [33], we decided to determine whether differences in NET formation can be related to DNAse activities of the *S. aureus* CF isolates. DNAse activities were measured using two independent methods, a fluorescence-based enzymatic activity protocol and an agar plate-based assay. As results in Figure 8A,B show, DNAse activities of the utilized *S. aureus* isolates spread across a range. Data obtained via the two independent methods strongly correlated with each other (Figure 8C). There was, however, no correlation between NET release induced by *S. aureus* isolates and their nuclease activity, suggesting that the DNAse activity expressed by the *S. aureus* isolates does not influence the amount of detectable NETs released from PMNs in vitro (Figure 8D,E).

## 4. Conclusions

We established an in vitro model to mimic the CF airway environment to study PMN effector functions. We hypothesized that PMNs from healthy individuals are capable of killing *S. aureus* isolates from CF patients, but bacterial killing would be inhibited by the CF airway environment. Our study aids to show that this simple, in vitro model mimics the impaired killing of *S. aureus* by PMNs observed in the airways of CF patients.

Chronic polymicrobial infections and PMN recruitment to the airways contribute to progressive lung disease in CF [34]. *S. aureus* is the most prevalent airway pathogen in CF and its resistance to antibiotics is increasing among adult patients [2]. PMNs are the first responders to the airways to fight infection; however, PMNs in the CF lung are inefficient at clearing *S. aureus*, leading to chronic infections. Therefore, understanding why PMNs are defective in combating *S. aureus* infections in CF is relevant to potentially reduce disease morbidity and mortality.

First, we showed that the CF sputum supernatant pretreatment does not affect the integrity of the PMN cell membrane. Therefore, the effect of inhibition on bacterial killing is not due to the loss of PMN cell viability. This was important to establish as normal PMN effector functions require live PMNs with intact plasma membrane [2]. Even PMN cytoplasts, ghost cells generated from human peripheral PMNs by experimental removal of their nuclei and most of their granules, are capable of motility, superoxide production and phagocytosis and maintain an intact plasma membrane. 

The “sputum cocktail” is a mixture of sputum supernatants from patients to demonstrate that this effect occurs across the board in CF disease. Individual sputum supernatants will be tested in the future using this model that could help further explain the variability amongst CF patients with regard to their PMNs’ impaired *S. aureus* killing. Elborn et al., also observed an upregulation of matrix metalloproteinase-8, annexin I, and nicotinamide phosphoribosyl transferase in CF sputum compared to healthy controls which has been associated with delayed apoptosis and clearing of inflammatory cells [35,36,37,38]. There is evidence to suggest that IL-8 and myeloperoxidase (MPO) are biomarkers in the CF sputum at different stages of lung inflammation, such that IL-8 correlates with exacerbations, and MPO and IgG degradation correlates with lower FEV_1_% score [39]. Disruption of the PMN cytoskeleton, delayed apoptosis, and upregulated cytokines in the CF sputum could all alter PMNs’ function in the lungs [40]. Understanding and further investigating these pathways using this in vitro model could lead to a novel discovery of a critical dysregulated pathway for PMNs to efficiently kill bacteria in the lungs of CF patients. 

A clinically relevant question is whether the inhibitory effect of human sputum on *S. aureus* killing by PMNs described here is unique to CF. Sputum obtained from healthy controls did not show any inhibition. While these results suggest that it is a specific feature of the CF sputum, more studies are needed to further confirm this as only a limited number of non-CF control subjects could be studied in this work.

PMNs in CF airways have been shown to co-localize with some bacteria (most likely phagocytosed) but CF blood PMNs were suggested to be impaired in intracellular bacterial killing [41,42,43]. Studies suggest that CFTR dysfunction may affect chloride supply to the phagolysosomes and impaired hypochlorous acid production, impacting NADPH oxidase and MPO-chlorinating activities [41,42,43,44]. A caveat to these studies is that bacterial killing was measured with CF blood PMNs and PMNs in the airways were not examined, which is where chronic infection takes place. Only very sporadic data are reported related to *P. aeruginosa* phagocytosis or killing by CF airway PMNs or PMNs under CF airway-like conditions [45,46]. Interestingly, our data show unimpaired phagocytosis of *S. aureus* when PMNs are exposed to the CF airway environment. While this assay is a combined measure of bacterial uptake and phagosomal pH changes, and the results suggest that phagocytosis of *S. aureus* by PMNs and subsequent phagosomal acidification is not affected by the CF airway environment, it has only been investigated in our study using one of the eight *S. aureus* isolates. Our data showed no difference in the overall ROS production between sputum-treated and untreated PMNs that is in contrast with a clinical study showing that sputum PMNs in CF patients display reduced respiratory burst [47]. Assessing the oxidative output in PMNs in the CF airways is a complex task that can partially depend on the experimental and detection systems used [48,49]. Superoxide production initiated by opsonized zymosan particles was not impaired by the CF sputum pretreatment of PMNs. This further confirms that the assembly and activation of the NADPH oxidase is not affected by the CF sputum pretreatment. As previously mentioned, the CFTR mutation has been proposed to impair chloride supply to the phagolysosomes and to affect PMN killing mechanisms in the phagosome [50]. A prior study reported that CF heterozygous PMNs have increased MPO activity [50]. While this could indeed have, theoretically, manifested in some of the variability in our control subject cohort, the same study reported that CF heterozygous PMNs had normal NADPH oxidase activity, MPO release and their microbial killing was directly not assessed. Therefore, due to these and the relatively rare occurrence of CFTR heterozygous patients in the normal population (1:25), it is unlikely to change the major conclusions in this work. 

Parts of the CF lung are nutrient rich and optimal for *S. aureus* growth. Based on a transcriptomic analysis of *S. aureus* grown in sputum, *S. aureus* can take up oxygen and is able to acquire iron in the CF lung [51]. *S. aureus* cultures isolated from the CF lung expressed virulence and metabolism genes that differed from *S. aureus* isolated from human joint infection and chronic wound infection [51]. The *S. aureus* isolates had a distinct metabolic profile, and it has been shown that metabolic status of bacteria impacts the efficacy of antibiotics, as well [51,52]. Our study also displays that the *S. aureus* isolates express DNAse I activity suggesting an evasion of NET-mediated killing by the bacterium. Although, NET release is not inhibited by the CF sputum, the bacterial isolates express DNAse activity to degrade the NETs being released and these data suggest that NETs being released by PMNs in the CF lung are ineffective in killing *S. aureus* and contribute to lung tissue damage. It has been shown that DNAse expression by *Streptococcus* allows the bacterium to escape NET-mediated killing by degradation, as the NETs were able to trap bacteria but not kill them [53,54,55]. DNAse I is an endonuclease that cleaves DNA phosphodiester bonds, targeting single-stranded DNA, double-stranded DNA, and chromatin DNA in a non-specific manner and has been used as a therapeutic for CF patients to degrade DNA and reduce sputum viscosity [56]. Therefore, patients prescribed DNAse I may display more resistance to NET-mediated bacterial killing. Altogether, PMNs in the CF lung generate NETs and ROS, however the CF sputum along with the virulent *S. aureus* affects the PMNs’ ability to efficiently kill. Further analysis of whole-genome sequences for all the *S. aureus* clinical isolates used in this study may help to interpret the discrepancies seen [30]. *S. aureus* is known to express multidrug-resistant genes and efflux transporters that are able to pump out toxic molecules [57,58]. The *S. aureus* resistance to killing is supported by the DNAse I activity expressed by the CF isolates in this study and a study that identified *S. aureus* CF clinical isolates display high nuclease activity that is protective against NET-mediated killing [33]. An abundance of NETs in the CF sputum correlated with chronic *S. aureus* infection in CF patients, suggesting that *S. aureus* may induce NET release by PMNs in the airways but deploy high nuclease activity to evade NET-mediated killing [33]. This abundance of NETs in the sputum is characteristic of the CF lung. Measurements of free DNA in CF patients’ sputum, an indirect indicator of NET release in the airway, show an association of declined lung function with increased NET presence in the lung [59]. The increased NET release from sputum-treated PMNs exposed to some of the *S. aureus* isolates is consistent with this increased NET presence in the CF lung.

This is the first study to investigate the capacity of PMNs to kill *S. aureus* CF clinical isolates. Our data demonstrate that *S. aureus* killing by PMNs is decreased by prior exposure to CF sputum. Attachment of bacteria to PMNs, their uptake into PMNs and PMN-mediated ROS production and NET formation are, however, not inhibited. Several other potential mechanisms could hide behind these observations that will be further investigated in the future. Steps of the PMN oxidative cascade downstream of the NADPH oxidase could be inhibited by the sputum environment. Patients who suffer from the inherited disorder, chronic granulomatous disease, characterized by mutations in proteins of the NADPH oxidase enzyme complex, are susceptible to *S. aureus* infections [10,60,61,62]. Published data suggest that downstream ROS such as MPO-derived HOCl are mainly responsible for direct killing of *S. aureus* and not initial members of the oxidative cascade, superoxide or H_2_O_2_ [10,60,61,62]. Inhibition of MPO enzymatic activity could lead to lower HOCl production in the phagolysosome and to impaired intracellular bacterial killing, in presence of unaltered phagocytosis and NADPH oxidase activity. The somewhat enhanced NET formation induced by the CF sputum detected in case of some of the *S. aureus* isolates could also lead to depriving critical components of the intracellular killing machinery, such as MPO and neutrophil elastase. Decreased primary granule fusion with the phagosome could clearly deliver suboptimal amounts of MPO and elastase leading to diminished bacterial killing [63,64]. *S. aureus* has been described to survive in PMN phagosomes and even lyse human PMNs in vitro [65,66]. While the CF sputum treatment alone does not affect PMN viability, it could prime PMNs for an enhanced or accelerated lysis by subsequent challenge by *S. aureus* that would ultimately result in impaired bacterial killing. 

Overall, our study demonstrates the usefulness of a novel, in vitro experimental model to mimic the CF airway environment and its effect on the antimicrobial effector functions of PMNs. We are the first to investigate bacterial killing of several *S. aureus* CF clinical isolates by human PMNs and to identify the difference in PMN killing of *S. aureus* clinical isolates following PMN exposure to CF and healthy sputum. The data suggest a CF sputum-dependent inhibition of PMN killing of *S. aureus.* This is the first study to. This model will be important for future studies elucidating the dysfunction of PMNs present in the airways of CF patients. 

## Figures and Tables

**Table 1:** *S. aureus* clinical isolates and strains used in this study.

Identifier in This Work	CF Sample Name	Methicillin Sensitivity	Strains or Isolates	CF Patient ID	References
MRSA24	CFBRSa24	resistant		CFBR-219	*Bernardy* et al. [22]
MRSA25	CFBRSa25	resistant		CFBR-134	
MRSA47	CFBRSa47	resistant	Cystic	CFBR-105	
MRSA74	CFBRSa74	resistant	Fibrosis	CFBR-201	
MSSA17	CFBR_EB_Sa117	sensitive	clinical	CFBR-280	
MSSA19	CFBR_EB_Sa119	sensitive	isolates	CFBR-309	
MSSA22	CFBR_EB_Sa122	sensitive		CFBR-322	
MSSA70	CFBRSa70	sensitive		CFBR-171	
USA 300	-	resistant	Reference strain	-	*S. aureus* subsp. aureus (ATCC BAA1717^TM^)
SA-rfp	-	-	JE2 background	-	This work

## Data Availability

The data presented in this study are available on request from the corresponding author.

## Abbreviations

PMN	polymorphonuclear neutrophil granulocyte
CF	cystic fibrosis
NET	neutrophil extracellular traps
MOI	multiplicity of infection
MRSA	methicillin-resistant *S. aureus*
MSSA	methicillin-sensitive *S. aureus*

## References

[1] Ahlgren H.G., Benedetti A., Landry J.S., Bernier J., Matouk E., Radzioch D., Lands L.C., Rousseau S., Nguyen D. (2015). Clinical outcomes associated with Staphylococcus aureus and Pseudomonas aeruginosa airway infections in adult cystic fibrosis patients. BMC Pulm. Med..

[2] Cystic Fibrosis Foundation Patient Registry (2018). 2017 Annual Data Report.

[3] Akil N., Muhlebach M.S. (2018). Biology and management of methicillin resistant Staphylococcus aureusin cystic fibrosis. Pediatr. Pulmonol..

[4] Lo D.K., Muhlebach M.S., Smyth A.R. (2018). Interventions for the eradication of meticillin-resistant Staphylococcus aureus (MRSA) in people with cystic fibrosis. Cochrane Database Syst. Rev..

[5] Muhlebach M.S., Zorn B.T., Esther C.R., Hatch J.E., Murray C.P., Turkovic L., Ranganathan S.C., Boucher R.C., Stick S.M., Wolfgang M.C. (2018). Initial acquisition and succession of the cystic fibrosis lung microbiome is associated with disease progression in infants and preschool children. PLoS Pathog..

[6] Dasenbrook E.C., Checkley W., Merlo C.A., Konstan M.W., Lechtzin N., Boyle M.P. (2010). Association between respiratory tract methicillin-resistant Staphylococcus aureus and survival in cystic fibrosis. JAMA J. Am. Med. Assoc..

[7] Ren C.L., Morgan W.J., Konstan M.W., Schechter M.S., Wagener J.S., Fisher K.A., Regelmann W.E., for The Investigators and Coordinators of the Epidemiologic Study of Cystic Fibrosis (2007). Presence of methicillin resistantStaphylococcus aureus in respiratory cultures from cystic fibrosis patients is associated with lower lung function. Pediatr. Pulmonol..

[8] Takei H., Araki A., Watanabe H., Ichinose A., Sendo F. (1996). Rapid killing of human neutrophils by the potent activator phorbol 12-myristate 13-acetate (PMA) accompanied by changes different from typical apoptosis or necrosis. J. Leukoc. Biol..

[9] Brinkmann V., Reichard U., Goosmann C., Fauler B., Uhlemann Y., Weiss D.S., Weinrauch Y., Zychlinsky A. (2004). Neutrophil Extracellular Traps Kill Bacteria. Science.

[10] De Jong N.W.M., Van Kessel K.P.M., Van Strijp J.A.G. (2019). Immune Evasion by Staphylococcus aureus. Microbiol. Spectr..

[11] Nasser A., Moradi M., Jazireian P., Safari H., Alizadeh-Sani M., Pourmand M.R., Azimi T. (2019). Staphylococcus aureus versus neutrophil: Scrutiny of ancient combat. Microb. Pathog..

[12] Liu Q., Mazhar M., Miller L.S. (2018). Immune and Inflammatory Reponses to Staphylococcus aureus Skin Infections. Curr. Dermatol. Rep..

[13] Harrison C.J. (2009). Innate immunity as a key element in host defense against methicillin resistant Staphylococcus aureus. Minerva Pediatr..

[14] Rawat A., Bhattad S., Singh S. (2016). Chronic Granulomatous Disease. Indian J. Pediatr..

[15] Kahl B.C., Becker K., Löffler B. (2016). Clinical significance and pathogenesis of staphylococcal small colony variants in persistent infections. Clin. Microbiol. Rev..

[16] Tirouvanziam R., Gernez Y., Conrad C.K., Moss R.B., Schrijver I., Dunn C.E., Davies Z.A., Herzenberg L.A. (2008). Profound functional and signaling changes in viable inflammatory neutrophils homing to cystic fibrosis airways. Proc. Natl. Acad. Sci. USA.

[17] Highlander S.K., Hultén K.G., Qin X., Jiang H., Yerrapragada S., O Mason E., Shang Y., Williams T.M., Fortunov R.M., Liu Y. (2007). Subtle genetic changes enhance virulence of methicillin resistant and sensitive Staphylococcus aureus. BMC Microbiol..

[18] Monecke S., Coombs G., Shore A.C., Coleman D.C., Akpaka P.E., Borg M., Chow H., Ip M., Jatzwauk L., Jonas D. (2011). A field guide to pandemic, epidemic and sporadic clones of methicillin-resistant staphylococcus aureus. PLoS ONE.

[19] Ibberson C.B., Parlet C.P., Kwiecinski J., Crosby H.A., Meyerholz D., Horswill A.R. (2016). Hyaluronan modulation impacts staphylococcus aureus biofilm infection. Infect. Immun..

[20] Nair D., Memmi G., Hernandez D., Bard J., Beaume M., Gill S., Francois P., Cheung A.L. (2011). Whole-genome sequencing of staphylococcus aureus strain rn4220, a key laboratory strain used in virulence research, identifies mutations that affect not only virulence factors but also the fitness of the strain. J. Bacteriol..

[21] Löfblom J., Kronqvist N., Uhlen M., Stahl S., Wernerus H. (2007). Optimization of electroporation-mediated transformation: Staphylococcus carnosus as model organism. J. Appl. Microbiol..

[22] Bernardy E.E., Petit R.A., Raghuram V., Alexander A.M., Read T.D., Goldberg J.B. (2020). Genotypic and phenotypic diversity of staphylococcus aureus isolates from cystic fibrosis patient lung infections and their interactions with pseudomonas aeruginosa. mBio.

[23] Yoo D.-G., Winn M., Pang L., Moskowitz S.M., Malech H.L., Leto T.L., Rada B. (2014). Release of Cystic Fibrosis Airway Inflammatory Markers fromPseudomonas aeruginosa–Stimulated Human Neutrophils Involves NADPH Oxidase-Dependent Extracellular DNA Trap Formation. J. Immunol..

[24] Rada B.K., Geiszt M., Káldi K., Timár C., Ligeti E. (2004). Dual role of phagocytic NADPH oxidase in bacterial killing. Blood.

[25] Pang L., Hayes C.P., Buac K., Yoo D.-G., Rada B. (2013). Pseudogout-Associated Inflammatory Calcium Pyrophosphate Dihydrate Microcrystals Induce Formation of Neutrophil Extracellular Traps. J. Immunol..

[26] Rada B., Jendrysik M.A., Pang L., Hayes C.P., Yoo D.-G., Park J.J., Moskowitz S.M., Malech H.L., Leto T.L. (2013). Pyocyanin-Enhanced Neutrophil Extracellular Trap Formation Requires the NADPH Oxidase. PLoS ONE.

[27] Yoo D.-G., Floyd M., Winn M., Moskowitz S.M., Rada B. (2014). NET formation induced by Pseudomonas aeruginosa cystic fibrosis isolates measured as release of myeloperoxidase–DNA and neutrophil elastase–DNA complexes. Immunol. Lett..

[28] Femling J.K., Cherny V.V., Morgan D., Rada B., Davis A.P., Czirják G., Enyedi P., England S.K., Moreland J.G., Ligeti E. (2006). The antibacterial activity of human neutrophils and eosinophils requires proton channels but not bk channels. J. Gen. Physiol..

[29] Sil P., Yoo D.-G., Floyd M., Gingerich A., Rada B. (2016). High throughput measurement of extracellular dna release and quantitative net formation in human neutrophils in vitro. J. Vis. Exp..

[30] Bernardy E.E., Petit R.A., Moller A.G., Blumenthal J.A., McAdam A.J., Priebe G.P., Chande A.T., Rishishwar L., Jordan I.K., Read T.D. (2019). Whole-Genome Sequences of Staphylococcus aureus Isolates from Cystic Fibrosis Lung Infections. Microbiol. Resour. Announc..

[31] Buvelot H., Posfay-Barbe K.M., Linder P., Schrenzel J., Krause K.-H. (2016). Staphylococcus aureus, phagocyte NADPH oxidase and chronic granulomatous disease. FEMS Microbiol. Rev..

[32] Martínez-Alemán S.R., Campos-García L., Palma-Nicolas J.P., Hernández-Bello R., González G.M., Sánchez-González A. (2017). Understanding the entanglement: Neutrophil extracellular traps (nets) in cystic fibrosis. Front. Cell. Infect. Microbiol..

[33] Herzog S., Dach F., De Buhr N., Niemann S., Schlagowski J., Chaves-Moreno D., Neumann C., Goretzko J., Schwierzeck V., Mellmann A. (2019). High Nuclease Activity of Long Persisting Staphylococcus aureus Isolates Within the Airways of Cystic Fibrosis Patients Protects Against NET-Mediated Killing. Front. Immunol..

[34] Cystic Fibrosis Foundation Patient Registry (2016). 2015 Annual Data Report.

[35] Pattison S.H., Gibson D.S., Johnston E., Peacock S., Rivera K., Tunney M., Pappin D.J., Elborn J.S. (2017). Proteomic profile of cystic fibrosis sputum cells in adults chronically infected with Pseudomonas aeruginosa. Eur. Respir. J..

[36] Jia S.H., Li Y., Parodo J., Kapus A., Fan L., Rotstein O.D., Marshall J.C. (2004). Pre–B cell colony–enhancing factor inhibits neutrophil apoptosis in experimental inflammation and clinical sepsis. J. Clin. Investig..

[37] Scannell M., Flanagan M.B., Destefani A., Wynne K., Cagney G., Godson C., Maderna P. (2007). Annexin-1 and Peptide Derivatives Are Released by Apoptotic Cells and Stimulate Phagocytosis of Apoptotic Neutrophils by Macrophages. J. Immunol..

[38] Gueders M.M., Balbin M., Rocks N., Foidart J.-M., Gosset P., Louis R., Shapiro S., Lopez-Otin C., Noël A., Cataldo D.D. (2005). Matrix Metalloproteinase-8 Deficiency Promotes Granulocytic Allergen-Induced Airway Inflammation. J. Immunol..

[39] Sloane A.J., Lindner R.A., Prasad S.S., Sebastian L.T., Pedersen S.K., Robinson M., Bye P.T., Nielson D.W., Harry J.L. (2005). Proteomic analysis of sputum from adults and children with cystic fibrosis and from control subjects. Am. J. Respir. Crit. Care Med..

[40] Castellani S., Di Gioia S., Di Toma L., Conese M. (2018). Human Cellular Models for the Investigation of Lung Inflammation and mucus production in cystic fibrosis. Anal. Cell. Pathol..

[41] Painter R.G., Valentine V.G., Lanson N.A., Leidal K., Zhang Q., Lombard G., Thompson C., Viswanathan A., Nauseef W.M., Wang G. (2006). CFTR expression in human neutrophils and the phagolysosomal chlorination defect in cystic fibrosis. Biochemistry.

[42] Dickerhof N., Isles V., Pattemore P., Hampton M.B., Kettle A.J. (2019). Exposure of Pseudomonas aeruginosa to bactericidal hypochlorous acid during neutrophil phagocytosis is compromised in cystic fibrosis. J. Biol. Chem..

[43] Painter R.G., Bonvillain R.W., Valentine V.G., Lombard G.A., LaPlace S.G., Nauseef W.M., Wang G. (2008). The role of chloride anion and CFTR in killing of Pseudomonas aeruginosa by normal and CF neutrophils. J. Leukoc. Biol..

[44] Hampton M.B., Kettle A.J., Winterbourn C.C. (1996). Involvement of superoxide and myeloperoxidase in oxygen-dependent killing of Staphylococcus aureus by neutrophils. Infect. Immun..

[45] Morris M.R., Doull I.J.M., Dewitt S., Hallett M.B. (2005). Reduced iC3b-mediated phagocytotic capacity of pulmonary neutrophils in cystic fibrosis. Clin. Exp. Immunol..

[46] Forrest O.A., Ingersoll S.A., Preininger M.K., Laval J., Limoli D.H., Brown M.R., Lee F.E., Bedi B., Sadikot R.T., Goldberg J.B. (2018). Frontline Science: Pathological conditioning of human neutrophils recruited to the airway milieu in cystic fibrosis. J. Leukoc. Biol..

[47] Houston N., Stewart N., Smith D.S., Bell S., Champion A.C., Reid D. (2013). Sputum neutrophils in cystic fibrosis patients display a reduced respiratory burst. J. Cyst. Fibros..

[48] Mittal M., Siddiqui M.R., Tran K., Reddy S.P., Malik A.B. (2014). Reactive Oxygen Species in Inflammation and Tissue Injury. Antioxid. Redox Signal..

[49] Guerra F.E., Addison C.B., De Jong N.W.M., Azzolino J., Pallister K.B., Van Strijp J. (2016). Staphylococcus aureus SaeR/S-regulated factors reduce human neutrophil reactive oxygen species production. J. Leukoc. Biol..

[50] Witko-Sarsat V., Allen R.C., Paulais M., Nguyen A.T., Bessou G., Lenoir G., Descamps-Latscha B. (1996). Disturbed Myeloperoxidase-Dependent Activity of Neutrophils in Cystic Fibrosis Homozygotes and Heterozygotes, and Its Correction by Amiloride. J. Immunol..

[51] Ibberson C.B., Whiteley M. (2019). TheStaphylococcus aureustranscriptome during cystic fibrosis lung infection. mBio.

[52] Lopatkin A.J., Stokes J.M., Zheng E.J., Yang J.H., Takahashi M.K., You L., Collins J.J. (2019). Bacterial metabolic state more accurately predicts antibiotic lethality than growth rate. Nat. Microbiol..

[53] Beiter K., Wartha F., Albiger B., Normark S., Zychlinsky A., Henriques-Normark B. (2006). an endonuclease allows streptococcus pneumoniae to escape from neutrophil extracellular traps. Curr. Biol..

[54] Walker M.J., Hollands A., Sanderson-Smith M.L., Cole J.N., Kirk J.K., Henningham A., McArthur J.D., Dinkla K., Aziz R., Kansal R.G. (2007). DNase Sda1 provides selection pressure for a switch to invasive group A streptococcal infection. Nat. Med..

[55] Buchanan J.T., Simpson A.J., Aziz R., Liu G.Y., Kristian S.A., Kotb M., Feramisco J., Nizet V. (2006). DNase expression allows the pathogen group a streptococcus to escape killing in neutrophil extracellular traps. Curr. Biol..

[56] Pressler T. (2008). Review of recombinant human deoxyribonuclease (rhDNase) in the management of patients with cystic fibrosis. Biol. Targets Ther..

[57] Jang S. (2016). Multidrug efflux pumps in Staphylococcus aureus and their clinical implications. J. Microbiol..

[58] Kaatz G.W., McAleese F., Seo S.M., Petersen P., Ruzin A., Dunman P.M., Murphy E., Projan S.J., Bradford P.A. (2005). Multidrug resistance in staphylococcus aureus due to overexpression of a novel multidrug and toxin extrusion (mate) transport protein. Antimicrob. Agents Chemother..

[59] Marcos V., Zhou-Suckow Z., Yildirim A., Önder Yildirim A., Bohla A., Hector A., Vitkov L., Krautgartner W.D., Stoiber W., Griese M. (2015). Free DNA in cystic fibrosis airway fluids correlates with airflow obstruction. Mediat. Inflamm..

[60] Pincus S.H., Klebanoff S.J. (1971). Quantitative Leukocyte Iodination. N. Engl. J. Med..

[61] Klebanoff S.J., Kettle A.J., Rosen H., Winterbourn C.C., Nauseef W.M. (2013). Myeloperoxidase: A front-line defender against phagocytosed microorganisms. J. Leukoc. Biol..

[62] Chapman A.L.P., Hampton M.B., Senthilmohan R., Winterbourn C.C., Kettle A.J. (2002). Chlorination of bacterial and neutrophil proteins during phagocytosis and killing of staphylococcus aureus. J. Biol. Chem..

[63] Metzler K.D., Goosmann C., Lubojemska A., Zychlinsky A., Papayannopoulos V. (2014). A Myeloperoxidase-containing complex regulates neutrophil elastase release and actin dynamics during NETosis. Cell Rep..

[64] Branzk N., Lubojemska A., Hardison S.E., Wang Q., Gutierrez M.G., Brown G.D., Papayannopoulos V. (2014). Neutrophils sense microbe size and selectively release neutrophil extracellular traps in response to large pathogens. Nat. Immunol..

[65] Voyich J.M., Braughton K.R., Sturdevant D.E., Whitney A.R., Saïd-Salim B., Porcella S.F., Long R.D., Dorward D.W., Gardner D.J., Kreiswirth B.N. (2005). Insights into Mechanisms Used byStaphylococcus aureusto Avoid Destruction by Human Neutrophils. J. Immunol..

[66] Rogers R., Tompsett R. (1952). The survival of staphylococci within human leucocytes. Bull. N. Y. Acad. Med..

